# The Continuing Challenges of Translational Research: Clinician-Scientists' Perspective

**DOI:** 10.1155/2012/246710

**Published:** 2012-08-09

**Authors:** Shervanthi Homer-Vanniasinkam, Janice Tsui

**Affiliations:** ^1^Leeds Vascular Institute, Leeds General Infirmary, Leeds LS1 3EX, UK; ^2^Division of Surgery and Interventional Science, University College London, Royal Free Campus, London NW3 2QG, UK

## Abstract

Over the last twenty years, revolutionary advances in biomedicine including gene therapy, stem cell research, proteomics, genomics and nanotechnology have highlighted the progressive need to restructure traditional approaches to basic and clinical research in order to facilitate the rapid, efficient integration and translation of these new technologies into novel effective therapeutics. Over the past ten years, funding bodies in the USA and UK such as the National Institutes of Health (NIH) and the Medical Research Council (MRC) have been driving translational research by defining and tackling the hurdles but more still remains to be achieved. This article discusses the ongoing challenges translational researchers face and outlines recent initiatives to tackle these including the new changes to translational funding schemes proposed by the NIH and the MRC and the launch of the “European Advanced Translational Research InfraStructure in Medicine” (EATRIS). It is anticipated that initiatives such as these will not only strengthen translational biomedical research programmes already initiated but should lead to rapid benefits to patients and society.

## 1. Introduction

Translational research (TR) is a relatively new area of investigation that ideally involves the integrated application of innovative technologies that encompass multiple disciplines including physiology, pathophysiology, natural history of disease, genetics, and proof-of-concept studies of drugs and devices [1].

TR describes a continuum of research in which basic science discoveries are utilized to prevent or treat human disease. It is an iterative process wherein scientific discoveries are integrated into clinical applications and, conversely, clinical observations are used to generate research foci for basic science: the “bench to bedside and back to bench” approach.

Initially, 2 phases in TR were described:

*T1*. basic science discoveries used to develop new treatments for disease (“bench to bedside”),

*T2*. research aimed at improving utilization of proven therapies in clinical practice and community settings (“bedside to community”).

More recently, this has been redefined to include 3 phases in TR, with the second phase being subdivided. Thus, in this new model T1 describes basic science to clinical science, T2 clinical science to clinical practice, and T3 is used to denote the translation of clinical practice to more widespread health improvements [2].

Whilst the importance and benefits of TR are now clear to most, significant challenges faced by investigators have also been recognised. This article focuses on some of those barriers and discusses whether initiatives over the past decade to drive TR forward as broken them down.

## 2. Barriers to Translational Research

The essential role of TR in ensuring rapid progression of basic scientific knowledge to patient benefit has been emphasised for more than 3 decades [3]. During this period, the barriers to TR have also been identified. The main issues that have been repeatedly debated include cultural differences between basic scientists and clinicians, lack of resources in terms of workforce and infrastructure, and the complex regulatory environment [4]. Cultural differences between the two groups of investigators largely stem from the lack of communication, differences in education and training, and different goals and reward mechanisms. Regulatory issues are intimidating to both basic scientists and clinicians and encompass ethics involved in human research, tissue banking and material transfer regulations, intellectual property rights, toxicology and manufacturing regulations, Food and Drug Administration (FDA) and Medicines and Healthcare Regulatory Agency (MHRA) approvals, study sponsorship and insurance, as well as trial and data monitoring. These are becoming even more complex with expanding work in the fields of cell and gene therapies and tissue engineering. Resource problems include lack of trained interdisciplinary staff to support investigations throughout the TR cycle, protected time for research particularly for clinicians, as well as access to shared resources. Together, these issues contribute to various checkpoints between the phases of TR including the “valley of death” that exists between preclinical research and clinical trials [5].

## 3. Streamlining Regulatory Issues

In addition to cultural differences, efforts have been made to streamline health research approvals and reduce regulatory burdens. The NIHR developed the Integrated Research Application System (IRAS) in 2008, an integrated online system which captures information required for approval application from several review bodies including the NHS R&D offices, research ethics committees, MHRA, Administration of Radioactive Substances Advisory Committee, Gene Therapy Advisory Committee, and the National Information Governance Board. The Research Passport system was also set up to streamline the issuing of honorary research contracts for researchers not employed by the NHS. In December 2011, the NHS launched the Health Research Authority which will play a key role in research governance and further streamline the approval process [6]. In the USA, the National Center for Advancing Translational Sciences (NCATS; http://www.ncats.nih.gov/) was established this year (2012) with its mission to “catalyze the generation of innovative methods and technologies that will enhance the development, testing, and implementation of diagnostics and therapeutics across a wide range of human diseases and conditions.” This would include dealing with bottlenecks such as regulatory issues in the TR process to make it “more efficient, less expensive and less risky” [7].

## 4. Training and Mentoring

Whilst regulatory issues are a major factor determining the success of translational projects, it is also imperative that we train and develop a pipeline of clinician-scientists and applied scientists who are comfortable in dealing with the continuing challenges of TR. During their training, clinician-scientists would benefit from experience in laboratory research whilst basic scientists should have exposure to the clinical environment under the guidance of experienced clinicians. Both groups should also receive training in research methodology including clinical trial design and conduct, and medical statistics. Multidisciplinary academic medical centres are well placed to offer such training programmes and indeed, the USA Clinical and Translational Sciences Awards (CTSA) scheme has as its central strategy five goals within which training and career development are firmly embedded [8].

*Goal 1*. Build national clinical and translational research capability.

*Goal 2*. Provide training and improving the career development of clinical and translational scientists.

*Goal 3*. Enhance consortium-wide collaborations.

*Goal 4*. Improve the health of our communities and the nation.

*Goal 5*. Advance T1 translational research.

Various M.D./M.B. Ph.D. programmes and master programmes focussing on TR are now also available and further training supported by dedicated fellowships are providing a clearer career pathway for both clinician-scientists and basic scientists.

Despite these encouraging schemes to entice and train young researchers in translational projects, there are still a number of challenges that need to be addressed if we are to ensure that the brightest minds will continue to enter this field of investigation. In clinical medicine, there are still some myths and misconceptions about the role of research in training programmes such as:

- research has no place in a clinician's training programme;
- there is no place for basic research in the clinical training programme;
- every surgical trainee should do some basic research;
- clinicians should only engage in clinical research.

These need to be tackled at faculty, institutional, and national levels.

Another challenge is that of career progression and promotion, and obtaining a tenured post in academic medicine or in a university science department. Current criteria for promotion still rely heavily on individual research output such as high impact publications, grants, and invited lectures. Investigators involved in TR may not be able to produce the required evidence of their contribution such as an adequate publication record, since translational projects generally take longer to complete. They are also likely to be part of a fairly large interdisciplinary team and their role in publications and on grants may not be apparent. Indeed the latter may be difficult to assess since they may be working outside their recognised disciplines. To improve this situation, institutions need to ensure that their tenure and promotions systems are able to evaluate and recognise the contributions investigators conducting TR make. Many institutions are working towards this, for example, by using reviewers with diverse expertise and understanding of TR and moving away from “traditional” assessment criteria.

The lack of role models and mentors in this area has been identified as a significant barrier by researchers trying to engage in TR. Mentorship is increasingly recognised as invaluable in the development of early career researchers into independent investigators and most institutions have in place mentoring programmes. In the UK, the Academy of Medical Sciences has an effective national mentoring and outreach scheme supported by the Department of Health and the NIHR which has been in place since 2002. This scheme specifically offers one-to-one mentoring for early career clinician-scientists by Academy Fellows who are leading scientists in their fields [9]. Further, the importance of training of effective mentors is also now recognised [10].

## 5. Continuing Problems

Despite increased efforts over the past decade, the effectiveness of these initiatives is still unclear. Outcomes of TR, particularly in the short term, have been difficult to measure, mainly due to the long timelines between discovery and clinical implementation. Traditional measures of research output such as contribution to high-impact publications may also be difficult to assess since TR publications usually involve multiple, interdisciplinary authors with varying roles and contributions. From the investigators' perspective, whilst there are examples of successful partnerships [11], recent discussions and publications continue to cite similar issues as barriers to TR across different specialties, both by basic scientists and by clinician-scientists. For example, in 2009, the NIH Task Force on Research in Emergency Medicine held a series of round-table discussions on emergency care research, with the aims of identifying key research questions in emergency care and barriers to research in emergency care [12–15]. The main barriers identified across the areas of emergency care were shortage of trained investigators, lack of role models and training opportunities, inadequate protected research time, poorly defined research-based career paths, the culture of valuing clinical care over research, poor infrastructure, lack of interdisciplinary research collaborations, lack of relevant funding streams, and ethical and regulatory issues. Of the recommendations made to address these issues, it was felt that formation and development of emergency care clinical research networks will be particularly beneficial to provide shared infrastructure and project support, as well as to increase patient accruals and trial size and improve efficiency [15]. The Federation of American Societies for Experimental Biology (FASEB) held a symposium entitled *Engaging Basic Scientists in Translational Research: Identifying Opportunities, Overcoming Obstacles* in March 2011 to identify and address issues important to basic scientists conducting TR [16]. The main challenges identified again included differences in culture and mindset between basic and clinical scientists, insufficient or nonsupportive infrastructure (including regulatory issues), difficulties developing and sustaining collaborations, inadequate training, insufficient funding, and lack of incentives and rewards. Recommendations were made as to how to tackle these hurdles, emphasizing the roles of institutions, professional societies, funding organisations, and individual scientists [17].

As discoveries move from “bench to bedside”, invaluable information may be gained by moving back from the “bedside to the bench”, for example, to understand important underlying mechanisms and unexpected findings and to make improvements. Similar barriers work against this arm of TR, such as poor communication of clinical challenges to basic scientists and lack of funding beyond the clinical trial. Unless researchers, be they clinical or scientific, can see “the light at the end of the (TR) tunnel,” we are likely to lose out on capturing, and captivating, high calibre translational researchers. Some of the TR initiatives are still in their early phases, so it is likely that further improvements will be seen in the near future.

## 6. Current Funding and Critical Issues: How Far Have We Come?

To overcome these barriers, increased sustained funding dedicated to TR is clearly essential to enable innovative approaches towards research to be developed. These include the building of research units that incorporate multidisciplinary groups which may involve bioinformaticians, statisticians, engineers, basic scientists, and clinicians; increasing expert support in regulatory issues and clinical trial design and conduct; as well as the initiation of forums for interdisciplinary discussion. The latter encourages discussion and networking between basic scientists and clinicians, initiation of interdisciplinary collaborations and the building of appropriate research teams.

In the USA and the UK, significant resources have already been directed towards TR via key initiatives. In the USA, the National Institutes of Health (NIH) launched the NIH Roadmap in 2003 aimed at supporting TR [18], followed by the Clinical and Translational Science Awards (CTSA) scheme in 2006 [8]. The CTSA scheme aimed to develop a consortium of institutes that would transform clinical and translational research; 60 academic medical centres are now funded, receiving USA$500 million annually. In the UK, the National Institute of Health Research (NIHR) spent *£*45 million to fund the first Biomedical Research Centres (BRC) and Biomedical Research Units (BRU) in 2007 and 2008 within NHS and university partnerships to drive translational research. A second round was launched in 2011, spending *£*775 million on 11 BRCs and 20 BRUs. BRCs support TR across a wide range of disease areas while BRUs are smaller groups working in priority areas including cardiovascular disease, dementia, hearing problems, nutrition and lifestyle, gastrointestinal, musculoskeletal, and respiratory disease [19]. Similarly, the MRC in a further expansion plan at the end of 2011 announced its commitment to a new *£*354m investment over the next 4 years. Specific calls were broadened to include stratified medicine research. This aims at understanding why groups of patients with the same diagnosis often differ in response to the same treatment and sets out to develop UK-wide research consortia each focussed on specific disease areas. The goal is to stratify disease processes and develop a clearer understanding of the underlying mechanisms that will eventually lead to future tailoring of treatment to individual patients and improve cost effectiveness. Additionally, the associated “Challenge Grants” will support ambitious, challenge-led studies of disease mechanisms in humans. These studies will produce major new mechanistic insights into human disease, with potential applicability to new therapeutic approaches and opportunities for “reverse translation” to more basic research. Other major funding bodies, including the Wellcome Trust, the Medical Research Council, the British Heart Foundation, and Heart Research UK, have also focussed support on TR. For example, Heart Research UK funded almost *£*500 K in 2010 on four projects under its Translational Research Grants programme [20] whilst the Wellcome Trust spent *£*59 million on TR in 2010 [21].

Nevertheless, with the growing realisation that the research infrastructure needed to cope with the broad spectrum of different diseases and treatment requirements cannot be comprehensively covered by any individual country alone, the beginning of 2012 also saw major commitments to TR widening and expanding with the initiation and launch of a new European research infrastructure initiative, the European Advanced Translational Research InfraStructure in Medicine (EATRIS).

Within this pan-European network of eleven major institutes across Europe, several governments and research bodies aim to ensure that the best research facilities for TR will be shared by the whole research community. Central broad areas for study include imaging and tracer development, biomarkers, vaccines, advanced therapy medicinal products, and infectious diseases.

In London, the new *£*73 million Imperial Centre for Translational and Experimental Medicine (ICTEM) will play a major role in the programme by combining laboratory space for 450 scientists including chemists, biologists, and engineers with dedicated facilities for assessing and developing novel medical therapies through clinical trials. Initial priority areas will include cancer, infection, cardiovascular, metabolic, and neurological diseases. The overall concept of the EATRIS programme is to bring together the best biomedical and clinical scientists and allow them access to state-of-the-art research facilities, techniques, and expertise covering the entire developmental chain from validation, compound libraries, good manufacturing practice, and so forth to research hospitals. Education is also a key element of the programme that will train scientists as well as technicians and nurses to think outside their immediate disciplines and provide information about clinical needs and regulatory requirements.

In the light of these initiatives, industry has also taken up the challenge of TR and funded projects independently, or in partnership with various funding bodies or with major academic health centres. A fine example of an industry-medical charity partnership is the MSD Wellcome Trust Hilleman Laboratories which were established in India in 2009, as a *£*90 million joint venture between MSD Laboratories India LLC (a wholly owned subsidiary of Merck & Co. Inc. USA) and the Wellcome Trust. The aim of this collaboration is to develop high-impact affordable vaccines for people in developing countries. Other companies have since followed suit. The MRC is investing *£*10 M in unique open innovation collaboration with AstraZeneca which has also formed partnerships with the Karolinska Institute to screen tissue banks for novel proteomic biomarkers that may signal underlying disease or susceptibility to risk. Similarly, Gentris Corporation has linked with academia in China to develop and validate new genomic markers while Affymetrix and Leica Microsystems are developing complementary companion diagnostic tests for personalized medicine by significantly increasing high through-put, *in situ* hybridization analyses. These partnerships provide academic researchers with unprecedented access to high-quality clinical and pre-clinical compounds, the building blocks of new drugs, in order to help better understand a spectrum of diseases with a view to exploring new treatments. Such collaboration have the potential to be transformational in stimulating relationships between academia and industry. The findings of the research will help deliver growth to the pharmaceutical and biotech industry.

## 7. Articles in the Current Issue

Despite concerns, there does seem to be a burgeoning of publications pertaining to TR. Thus, it is heartening to read in this special issue of Cardiology Research and Practice articles devoted to translational science and research. These papers address a range of cardiovascular diseases including peripheral arterial disease, abdominal aortic aneurysms, systemic sclerosis, and pulmonary artery hypertension, discussing recent findings on disease initiation and development as well as novel therapies that are currently being explored. They illustrate how improved knowledge of basic pathological processes and their regulatory mechanisms are contributing to our understanding of specific diseases. For example, Spirig et al. [22] and Patel et al. [23] discuss the increasingly recognised link between innate immune signalling and cardiovascular diseases whilst Williams et al. [24] and Dooley et al. [25] demonstrate the potential of modulating the ubiquitous nitric oxide pathway in the treatment of peripheral arterial disease and systemic sclerosis, respectively. The impact of advanced technologies on TR is clearly seen in the field of genetics and in this issue, Harrison and colleagues describe the use of genomics to study pathways involved in abdominal aortic aneurysm development [26] whist Caporali & Emanueli discuss the role of microRNAs in vascular repair [27]. In terms of novel therapies, contributions on remote ischaemia preconditioning [28], stem cell therapies [29], and tissue protective strategies [30] illustrate their potential in the cardiovascular field. This certainly indicates that TR is being pursued intensively by these investigators and departments, and in subjects that address areas of either unmet, or indeed, unrealized, clinical need. It is also encouraging to see that most of the papers in this issue come from multidisciplinary groups of clinicians and scientists at various stages of their careers working in close partnership. Collaborations such are these are the driving forces of TR in terms of bringing together expertise, integrating basic science and clinical applications, and training future generations of clinician-scientists.

## 8. Concluding Remarks

TR is not for the faint hearted. The constant challenges of teaching, researching, publishing, and competing for limited sources of funding, coupled with pursuing career aims and ambitions, can seem daunting. However, it can also be a deeply satisfying and exhilarating endeavour, especially when the fruits of the experimental laboratory are translated into improved healthcare delivery to our patients.

Finally, we feel that TR has a central and pivotal role in harnessing significant discoveries in biomedical science for the benefit of our patients. To the sceptics who ask: “Where is the evidence that TR matters?” we would answer: “As with Sir Christopher Wren's monuments, the evidence is all around us.” 
